# Associations between lipoprotein(a), oxidized phospholipids, and extracoronary vascular disease

**DOI:** 10.1016/j.jlr.2024.100585

**Published:** 2024-06-26

**Authors:** Tiffany R. Bellomo, Yuxi Liu, Thomas C. Gilliland, Hannah Miksenas, Sara Haidermota, Megan Wong, Xingdi Hu, Joaquim Rosado Cristino, Auris Browne, Jorge Plutzky, Sotirios Tsimikas, James L. Januzzi, Pradeep Natarajan

**Affiliations:** 1 Division of Vascular and Endovascular Surgery, Massachusetts General Hospital, Boston, MA, USA; 2 Division of Cardiology, Massachusetts General Hospital, Harvard Medical School, Boston, MA, USA; 3 Program in Medical and Population Genetics and the Cardiovascular Disease Initiative, Broad Institute of Harvard and MIT, Cambridge, MA, USA; 4 Novartis Pharmaceuticals Corporation, Novartis, East Hanover, NJ, USA; 5 Division of Cardiology, Brigham and Women’s Hospital, Boston, Harvard Medical School, Boston, MA, USA; 6 Sulpizio Cardiovascular Center, University of California San Diego, La Jolla, CA, USA; 7 Cardiology Division, Baim Institute for Clinical Research, Boston, MA, USA

**Keywords:** peripheral artery disease, lipoprotein(a), lipid, peripheral angiography

## Abstract

The roles of lipoprotein(a) [Lp(a)] and related oxidized phospholipids (OxPLs) in the development and progression of coronary disease is known, but their influence on extracoronary vascular disease is not well-established. We sought to evaluate associations between Lp(a), OxPL apolipoprotein B (OxPL-apoB), and apolipoprotein(a) (OxPL-apo(a)) with angiographic extracoronary vascular disease and incident major adverse limb events (MALEs). Four hundred forty-six participants who underwent coronary and/or peripheral angiography were followed up for a median of 3.7 years. Lp(a) and OxPLs were measured before angiography. Elevated Lp(a) was defined as ≥150 nmol/L. Elevated OxPL-apoB and OxPL-apo(a) were defined as greater than or equal to the 75th percentile (OxPL-apoB ≥8.2 nmol/L and OxPL-apo(a) ≥35.8 nmol/L, respectively). Elevated Lp(a) had a stronger association with the presence of extracoronary vascular disease compared to OxPLs and was minimally improved with the addition of OxPLs in multivariable models. Compared to participants with normal Lp(a) and OxPL concentrations, participants with elevated Lp(a) levels were twice as likely to experience a MALE (odds ratio: 2.14, 95% confidence interval: 1.03, 4.44), and the strength of the association as well as the C statistic of 0.82 was largely unchanged with the addition of OxPL-apoB and OxPL-apo(a). Elevated Lp(a) and OxPLs are risk factors for progression and complications of extracoronary vascular disease. However, the addition of OxPLs to Lp(a) does not provide additional information about risk of extracoronary vascular disease. Therefore, Lp(a) alone captures the risk profile of Lp(a), OxPL-apoB, and OxPL-apo(a) in the development and progression of atherosclerotic plaque in peripheral arteries.

## Graphical Abstract

**Figure:**
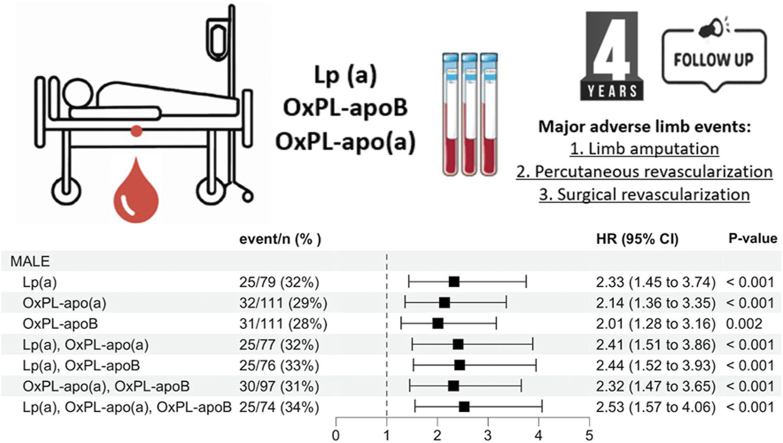

Vascular health beyond the coronary arteries is a worsening global public health issue (1, 2, 3) that affects over 200 million patients worldwide in the lower extremities alone (2). The incidence of extracoronary vascular disease is projected to increase with an aging population (1, 4, 5). Extracoronary vascular diseases are progressive chronic diseases characterized by arterial narrowing due to atherosclerotic plaque, which results in insufficient blood flow to the affected organ (6, 7). These diseases include lower extremity, carotid, subclavian, and renal arterial disease. While many patients experience a prolonged asymptomatic disease course, the diagnosis frequently manifests with symptoms at late stages when the risk for major adverse limb events (MALEs) is the greatest (8, 9, 10). The occurrence of MALE confers increased risk of mortality, with upward of 50% of patients dying within 1 year of amputation related to lower extremity extracoronary vascular disease (11). Better tools are needed to identify patients with extracoronary vascular disease at high risk of MALE and guide treatments to reduce risks for both incident and progressive disease.

Clinical practice guidelines for the treatment of extracoronary vascular disease have focused on targeting modifiable risk factors, with a primary emphasis on hyperlipidemia (12, 13, 14). Hyperlipidemia plays a central role in the development and progression of atherosclerosis, where elevated levels of low-density lipoprotein cholesterol (LDL-C) cause lipid deposition in arterial walls, inflammation, and plaque formation (15, 16). However, even after controlling LDL-C levels, major adverse cardiovascular events remain elevated among patients with extracoronary vascular disease. Patients with extracoronary vascular disease have higher risk of mortality than patients with coronary artery disease (CAD), even when the latter is treated with cholesterol lowering drugs (17, 18). Proatherogenic lipoprotein(a) [Lp(a)] is composed of apolipoprotein(a) (apo(a)) and apolipoprotein B (apoB) and is the primary plasma sink for oxidized phospholipids (OxPLs) (19, 20, 21, 22) Despite established associations between Lp(a) and risk of severe coronary artery atherosclerosis, the role of Lp(a) and OxPLs in development and progression of extracoronary vascular disease is controversial. Some studies have found elevated Lp(a) levels are associated with risk of developing lower extremity extracoronary vascular disease (20, 23, 24, 25, 26, 27, 28), while large prospective cohort studies have not identified this association (29, 30). Importantly, the role or predictive value of Lp(a) in identifying patients with extracoronary vascular disease at high risk of complications, including MALE, remains unknown.

In this study, we sought to investigate the association of Lp(a) and related OxPL with extracoronary vascular disease and risk of MALE. We utilized previously collected data from participants who underwent coronary and/or peripheral angiography in the Catheter Sample Blood Archive of Cardiovascular Diseases (CASABLANCA) study. We hypothesized that Lp(a) and OxPLs would be associated with prevalent angiographic extracoronary vascular disease and would provide prognostic information regarding future limb-related complications.

## Materials and methods

All study procedures were approved by the Massachusetts General Hospital Institutional Review Board and carried out in accordance with the Declaration of Helsinki. The design of the CASABLANCA study has been described previously (ClinicalTrials.gov Identifier: NCT00842868 (31). Briefly, 1251 participants undergoing coronary and/or peripheral angiography with or without intervention between 2008 and 2011 were prospectively enrolled at the Massachusetts General Hospital in Boston, MA, USA. Participants were enrolled in the study at the time of their index angiogram. Medical record review from time of enrollment to end of follow-up was performed. Median follow-up was 3.7 years (IQR 3.1, 4.3), ranging from a minimum of 1 day to a maximum 4.8 years. For identification of clinical endpoints, review of medical records as well as phone follow-up with participants and/or managing physicians was performed. A detailed definition of endpoints for CASABLANCA was previously published (31). MALE was defined as any limb amputation, open surgical revascularization, or percutaneous revascularization of noncoronary arterial beds. Participants with extracoronary vascular disease were followed up to incident MALE.

Specific to this analysis and in accordance with our previous methodology (32), we included a total of 446 participants (supplemental Fig. S1). There were 152 participants who underwent peripheral angiography, of which 141 participants had confirmed extracoronary vascular disease and 11 participants did not have extracoronary vascular disease. Thirteen participants underwent both peripheral and coronary angiography but had no angiographically significant CAD or extracoronary vascular disease. There were 281 participants underwent coronary angiography alone and had no angiographically significant CAD, no prior history of atherosclerotic disease, no symptoms of claudication, and no atherosclerotic events in follow-up. These participants were included to improve statistical power and classified as controls without significant peripheral obstruction, given their medical history and absence of extracoronary vascular disease diagnosis and/or complications during follow-up. Extracoronary vascular disease was defined as stenosis more than 50% in any peripheral artery, including lower extremity, carotid, subclavian, and renal arteries (33). The indications for peripheral angiography included claudication (n = 100), hypertension (n = 22), carotid artery stenosis with or without stroke (n = 11), and other extracoronary vascular disease (n = 30). Angiography was performed of the lower extremity arteries (n = 129), renal arteries (n = 59), and carotid/subclavian vessels (n = 18).

### Biomarker testing

Lp(a) was measured using a minor modification of a novel immunoassay using a biotin-modified LPA-KIV9 monoclonal antibody instead of horseradish peroxidase-modified LPA-KIV9 (34). The principle of the new assay is the capture of Lp(a) with monoclonal antibody LPA4 primarily directed to an epitope in apo(a) KIV2, and its detection with monoclonal antibody LPA-KIV9 directed to a single antigenic site present on KIV9. No statistically or clinically significant bias was observed between Lp(a) measurements obtained by the LPA4/LPA-KIV9 ELISA and those obtained by the gold-standard University of Washington assay, and therefore, the methods were previously determined to be equivalent (35). OxPLs associated with apoB and apo(a) were quantified via immunoassay using capture antibodies specific to apoB (MB47) and apo(a) (LPA4), followed by a secondary antibody EO6, a murine monoclonal antibody that specifically recognizes the phosphocholine headgroup of oxidized but not native phospholipids (36, 37). Units are reported as nmol/L of phosphocholine equivalents of phosphocholine-containing OxPLs.

Literature has extensively characterized the distributions and correlations of Lp(a) and OxPLs, demonstrating that Lp(a) is highly correlated with both OxPL-apo(a) and OxPL-apoB (38, 39). In order to redemonstrate these correlations within this cohort to understand the generalizability, a correlation heatmap was generated between each Lp(a)-related biomarker and traditional lipid parameters.

Elevated Lp(a) was defined as Lp(a) ≥150 nmol/L based on current clinical practice guidelines (40). Elevated OxPL-apoB and OxPL-apo(a) were defined as greater than or equal to the 75th percentile (OxPL-apoB ≥8.2 nmol/L and OxPL-apo(a) ≥35.8 nmol/L). Specifically, the data were divided in to four equal groups, each containing 25% of the total N. For OxPL-apoB, the value of the first quartile was 2.75, the value of the second quartile was 3.69, and the value for the third quartile was 8.17. For OxPL-apo(a), the value of the first quartile was 4.57, the value of the second quartile was 11.77, and the value for the third quartile was 35.79. Thresholds for each biomarker to discriminate extracoronary vascular disease confirmed the chosen definition for elevated biomarkers, as the optimal cutoff point was close to 150 nmol/L for Lp(a) and around the 75th percentile for OxPLs (supplemental Table S1).

Biomarkers were log_(2)_-transformed to create normal distributions for statistical analysis.

### Statistical analysis

Median (interquartile range) and count (frequency) were used to present continuous and categorical variables, respectively. Kruskal-Wallis and chi-square tests compared baseline characteristics in participants with and without extracoronary vascular disease. Logistic regressions were run to assess the association between Lp(a), OxPL-apoB, and OxPL-apo(a) with extracoronary vascular disease compared to participants without extracoronary vascular disease. Consistent with prior studies (27, 41), covariates included age, sex, race, systolic blood pressure, HDL-c, total cholesterol, diabetes, smoking, and history of cardiovascular disease.

We then restricted our cohort to participants only with extracoronary vascular disease, regardless of Lp(a) or OxPL levels. We conducted multivariable Weibull accelerated failure time regression analysis to assess the association between Lp(a) and incident MALE among participants with extracoronary vascular disease. Accelerated failure time models assume the effect of a relevant covariate (in this case Lp[a] and related particles) have an increasingly important effect over the life course of a disease rather than exerting a constant risk. The exponentiated Weibull regression coefficient is known as event time ratio (ETR). An ETR of 1.0 means no association between the independent variable and the outcome. Values lower than 1.0 indicate an earlier incidence of the outcome, and values higher than 1.0 indicate a delayed incidence of the outcome. In addition, hazard ratio (HR) per 1 unit increment in log_(2)_ particle concentration with 95% confidence intervals was expressed. These were graphed in a restricted cubic spline curve. We reported HRs of MALE stratified by high and normal level of both Lp(a) and OxPLs. Participants with normal levels for Lp(a) and OxPL were used as reference.

There was minimal missing data in this cohort, where the data field with the highest amount of missingness, HDL, only had 5.8% missing data fields. Before building logistic regression models and Cox models, MICE imputation was employed to ensure there was no missing data for model analysis. All *P*-values reported were 2-sided. A *P* value <0.05 was considered statistically significant. All statistical analyses were performed using R, version 4.2.2 (R Foundation for Statistical Computing, Vienna, Austria. URL: https://www.R-project.org/).

## Results

A total of 446 study participants were included in this study. The mean age of participants was 64 ± 11 years, and 247 (55.4%) were male (Table 1). Of these, 141 (31.6%) had extracoronary vascular disease at baseline. Those with angiographically demonstrated extracoronary vascular disease were older, more likely be male, and had higher prevalence of hypertension, dyslipidemia, CAD, prior myocardial infarction, diabetes mellitus, prior cerebrovascular accident, and chronic kidney disease. Of the participants with extracoronary vascular disease, 87% were prescribed statin medications, resulting in significantly lower total cholesterol (139 mg/dl vs. 167 mg/dl) and LDL-C (70 mg/dl vs. 99 mg/dl) levels compared to participants without extracoronary vascular disease. Participants with extracoronary vascular disease had higher levels of hemoglobin A1c, fasting glucose, creatinine, blood urea nitrogen, and Lp(a). Of the participants with extracoronary vascular disease, participants with elevated Lp(a) had higher prevalence of CAD, heart failure, myocardial infarction, chronic obstructive pulmonary disease, smoking, and history of coronary intervention compared to those with normal Lp(a) levels (supplemental Table S2). Within these participants, we observed a high degree of correlation between Lp(a) and OxPLs, with a statistically significant Spearman correlation coefficient of 0.90 for OxPL-apo(a) and 0.95 for OxPL-apoB (supplemental Fig. S2). To confirm this correlation in our cohort, we split participants into quartiles based on Lp(a) levels (supplemental Table S3). This table demonstrates there is a significant increase in the median OxPL-apo(a) and OxPL-apoB levels across increasing quartiles of Lp(a).

**Table 1:** Baseline characteristics of study patients

Demographics	Subjects Without Extracoronary Vascular Disease (n = 305)	Subjects With Extracoronary Vascular Disease (n = 141)	*P* Value
Age, year (mean (SD))	62.9 (11.5)	67.9 (11.1)	<0.001
Male (%)	175 (57.4)	99 (70.2)	0.01
Caucasian (%)	274 (89.8)	132 (93.6)	0.35
Medical conditions (%)			
Hypertension	188 (61.6)	132 (93.6)	<0.001
Dyslipidemia	154 (50.5)	106 (75.2)	<0.001
Coronary artery disease	71 (23.3)	90 (63.8)	<0.001
Prior MI	27 (8.9)	33 (23.4)	<0.001
Heart Failure	78 (25.6)	26 (18.4)	0.12
COPD	61 (20.0)	31 (22.0)	0.72
Diabetes Mellitus	51 (16.7)	58 (41.1)	<0.001
CVA/TIA	24 (7.9)	27 (19.1)	0.001
Chronic kidney disease	16 (5.2)	30 (21.3)	<0.001
Renal replacement therapy	4 (1.3)	3 (2.1)	0.81
Smoker	38 (12.6)	25 (18.0)	0.18
Afib/Aflutter	76 (24.9)	21 (14.9)	0.02
Prior angioplasty	14 (4.6)	46 (32.6)	<0.001
Prior CABG	11 (3.6)	30 (21.3)	<0.001
Prior PCI	39 (12.8)	46 (32.6)	<0.001
Medications (%)			
ACEi/ARB	142 (47.0)	90 (63.8)	0.001
β-blocker	170 (56.1)	96 (68.1)	0.02
Aldosterone antagonist	12 (4.0)	8 (5.7)	0.57
Loop diuretic	68 (22.4)	28 (19.9)	0.62
Nitrate	26 (8.6)	26 (18.4)	0.004
CCB	60 (19.8)	47 (33.6)	0.002
Statin	166 (54.8)	122 (86.5)	<0.001
Aspirin	186 (61.4)	123 (87.2)	<0.001
Warfarin	65 (21.5)	19 (13.6)	0.07
Clopidogrel	33 (10.9)	50 (35.5)	<0.001
Laboratory data (median [IQR]) [Range]			
Sodium, mEq/L	140 (138, 141) [128, 149]	140 (137, 141) [132, 148]	0.85
BUN, mg/dl	17 (14, 21) [5, 86]	21 (16, 28) [8, 67]	<0.001
Creatinine, mg/dl	1.00 (0.86, 1.20) [0.5, 11.67]	1.20 (0.98, 1.52) [0.67, 2.97]	<0.001
Glycohemoglobin, %	5.8 (5.4, 6.6) [4.4, 13.9]	6.8 (5.9, 9.0) [4.1, 12.4]	0.008
Glucose, mg/dl	99 (89, 116) [64, 342]	106 (93, 123) [61, 246]	0.02
Hemoglobin, g/dl	13.5 (12.2, 14.6) [9.6, 18.8]	13.0 (11.6, 14.0) [9.0, 18.2]	0.001
Total cholesterol, mg/dl	167 (137, 194) [93, 339]	139 (118, 170) [55, 277]	<0.001
LDL-C, mg/dl	95 (69, 118) [35, 222]	70 (56, 89) [16, 194]	<0.001
HDL-C, mg/dl	43 (35–54) [15, 152]	40 (32–50) [10, 104]	0.09
Triglycerides, mg/dl	110 (79–140) [38, 528]	110 (80–170) [27, 547]	0.78
Lp(a), nmol/L	24.61 (11.18, 74.17) [0.00, 599.58]	32.75 (11.15, 137.30) [1.52, 497.76]	0.08
OxPL-apo(a), nmol/L	10.47 (4.81, 31.34) [0.9, 77.7]	13.21 (4.48, 42.52) [1.09, 74.11]	0.19
OxPL-apoB, nmol/L	3.61 (2.78, 7.27) [0.9, 25.6]	3.98 (2.70, 10.84) [1.27, 31.99]	0.19
Biomarker Thresholds (%)			
Lp(a) >150 nmol/L	35 (11.5)	29 (20.6)	
OxPL-apo(a) >35.8 nmol/L	64 (21.0)	47 (33.3)	
OxPL-apoB >8.2 nmol/L	65 (21.3)	46 (32.6)	

ACEi, angiotensin converting enzyme inhibitor; Afib, atrial fibrillation; ARB, angiotensin receptor blocker; BUN, blood urea nitrogen; CABG, coronary artery bypass graft; CCB, calcium channel blocker; CVA, cerebrovascular accident; HDL-C, high density lipoprotein cholesterol; LDL-C, low density lipoprotein cholesterol; Lp(a), lipoprotein(a); MI, myocardial infarction; OxPL, oxidized phospholipid; PCI, percutaneous coronary intervention; TIA, transient ischemic event.

Table 2 details the association between Lp(a), OxPLs, and extracoronary vascular disease. In univariable models, elevated Lp(a) (odds ratio [OR]: 1.96, 95% confidence interval [CI]: 1.14, 3.36), OxPL-apo(a) (OR: 1.84, 95% CI: 1.18, 2.88), and combinations of these levels (OR: 1.96, 95% CI: 1.14, 3.36]) were significantly associated with increased risk of extracoronary vascular disease. All univariable models had a similar C-statistic ranging from 0.54 to 0.57. After adjustment for age, sex, race, systolic blood pressure, HDL-C, total cholesterol, diabetes, smoking, and history of cardiovascular disease, these associations remained significant: Lp(a) (OR: 2.14, 95% CI: 1.03, 4.44), OxPL-apo(a) (OR: 1.64, 95% CI: 0.92, 2.92), and Lp(a)&OxPL-apo(a) (OR: 2.14, 95% CI: 1.03, 4.44). Although elevated OxPL-apoB was significantly associated with extracoronary vascular disease in univariable modes, OxPL-apoB was not significantly associated with extracoronary vascular disease in multivariable models. Participants with elevated Lp(a)&OxPL-apo(a)& OxPL-apoB (OR: 2.35, 95% CI:1.12, 4.91) were at the highest risk of extracoronary vascular disease. Multivariate models for extracoronary vascular disease including covariates and Lp(a) had a C-statistic of 0.82, which was unchanged with the serial addition of OxPLs (supplemental Table S4). To assess the predictive power of OxPL in participants with low levels of Lp(a), we restricted participants to those with Lp(a) less than the 25^th^ percentile and found OxPL-apoB was no longer associated with extracoronary vascular disease (OR: 0.41, 95% CI:0.07, 2.16) (supplemental Table S5).

**Table 2:** Association of Lp(a) and oxidized phospholipids with extracoronary vascular disease

Biomarkers	Univariable Model			Multivariable Model		
	OR (95% CI)	*P* Value	C-Statistic	OR (95% CI)	*P* Value	C-Statistic
Lp(a)						
Log_(2)_, per 1 unit increment	1.16 (1.00, 1.35)	0.04	0.55	1.15 (0.97, 1.36)	0.11	0.82
Level ≥150 nmol/L	1.96 (1.14, 3.36)	0.01	0.54	2.14 (1.03, 4.44)	0.04	0.82
OxPL-apoB						
Log_(2)_, per 1 unit increment	1.36 (1.02, 1.81)	0.03	0.54	1.18 (0.83, 1.70)	0.36	0.81
Level ≥8.2 nmol/L	1.75 (1.12, 2.73)	0.01	0.55	1.24 (0.69, 2.17)	0.46	0.81
OxPL-apo(a)						
Log_(2)_, per 1 unit increment	1.11 (0.94, 1.33)	0.23	0.54	1.04 (0.84, 1.30)	0.72	0.81
Level ≥ 35.8 nmol/L	1.84 (1.18, 2.88)	0.007	0.56	1.64 (0.92, 2.92)	0.09	0.82
Combination of levels						
Lp(a)&OxPL-apoB	2.17 (1.25, 3.76)	0.006	0.55	2.35 (1.13, 4.91)	0.02	0.82
Lp(a)&OxPL-apo(a)	1.96 (1.14, 3.36)	0.01	0.54	2.14 (1.03, 4.44)	0.04	0.82
OxPL-apoB&OxPL-apo(a)	2.11 (1.33, 3.36)	0.002	0.57	1.74 (0.96, 3.18)	0.07	0.82
Lp(a)&OxPL-apoB&OxPL-apo(a)	2.17 (1.25, 3.76)	0.006	0.55	2.35 (1.12, 4.91)	0.02	0.82

Multivariable model: age, sex, race, systolic blood pressure, HDL-C, total cholesterol, diabetes, smoking and history of cardiovascular disease. apoB, apolipoprotein B; apo(a), apolipoprotein(a); CI, confidence interval; Lp(a), lipoprotein(a); OR, odds ratio; OxPL, oxidized phospholipids.

**Fig. 1:**
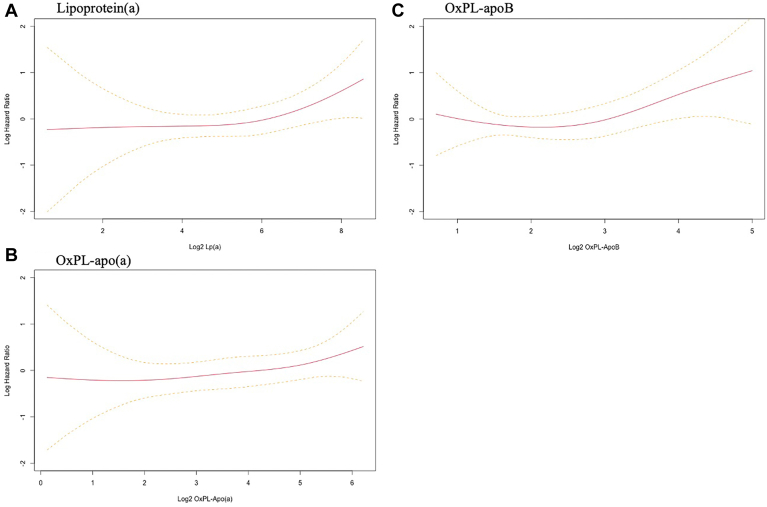
Restricted cubic spline curve showing the association between (A) log(2) Lp(a), (B) OxPL-apoB, and (C) OxPL-apo(a) levels with major adverse limb events.

Over median 3.7 years of follow-up, 91 cases of MALE occurred (13 cases of limb amputation, 22 surgical revascularization, and 56 percutaneous revascularization), and 38 (42%) participants had diabetes. Figure 1 depicts restricted cubic spline analyses for MALE by Lp(a) and OxPL levels. The graph demonstrates that higher Lp(a), OxPL-apoB, and OxPL-apo(a) levels were all associated with progressively elevated risk of MALE. The HR for MALE with dichotomous values is shown in Table 3, which also demonstrates a significant association between levels of Lp(a) and OxPLs. In a univariable model, participants with elevated Lp(a) (HR: 2.22, 95% CI: 1.34, 3.69), OxPL-apo(a) (HR: 2.14, 95% CI: 1.36, 3.35), OxPL-apoB (HR: 2.01, 95% CI: 1.28, 3.16), and combinations of all levels were twice as likely to experience a MALE. HRs were slightly attenuated after adjustment in a multivariable for aforementioned covariates, but participants with elevated OxPL-apo(a) were at highest risk of MALE (HR: 2.11, 95% CI: 1.28, 3.49). Multivariate models for MALE including covariates and Lp(a) had a C-statistic of 0.77, which decreased with the serial addition of OxPLs (supplemental Table S6). To assess the predictive power of OxPL in participants with low levels of Lp(a), we restricted participants to those with Lp(a) less than the 25^th^ percentile and found OxPL-apoB (OR: 0.67 and 95% CI:0.10, 4.42) and OxPL-apo(a) (OR: 1.18 and 95% CI:0.45, 3.06) were no longer associated with MALE (supplemental Table S5). A variance inflation factor was calculated within these models for both Lp(a) and OxPLs. The variance inflation factor of 9 for these models indicates the presence and high intensity of multicollinearity.

**Table 3:** Association of Lp(a) and oxidized phospholipids with major adverse limb event in patients with extracoronary vascular disease

Biomarkers	Univariable Model			Multivariable Model		
	HR (95% CI)	*P* Value	C-Statistic	HR (95% CI)	*P* Value	C-Statistic
Lp(a)						
Log_(2)_, per 1 unit increment	1.26 (1.06, 1.49)	0.008	0.58	1.21 (1.01, 1.45)	0.04	0.77
Level ≥150 nmol/L	2.22 (1.34, 3.69)	0.002	0.56	2.04 (1.14, 3.63)	0.02	0.77
OxPL-apoB						
Log_(2)_, per 1 unit increment	1.50 (1.11, 2.03)	0.008	0.57	1.37 (1.00, 1.89)	0.05	0.76
Level ≥8.2 nmol/L	2.01 (1.28, 3.16)	0.002	0.57	1.70 (1.03, 2.80)	0.04	0.76
OxPL-apo(a)						
Log_(2)_, per 1 unit increment	1.25 (1.02, 1.53)	0.003	0.58	1.21 (0.98, 1.50)	0.08	0.76
Level ≥35.8 nmol/L	2.14 (1.36, 3.35)	<0.001	0.58	2.11 (1.28, 3.49)	0.003	0.77
Combination of levels						
Lp(a)&OxPL-apoB	2.36 (1.42, 3.92)	<0.001	0.56	2.08 (1.17, 3.70)	0.01	0.77
Lp(a)&OxPL-apo(a)	2.22 (1.34, 3.69)	0.002	0.56	2.04 (1.14, 3.63)	0.02	0.77
OxPL-apoB&OxPL-apo(a)	2.32 (1.47, 3.65)	<0.001	0.58	2.07 (1.24, 3.44)	0.005	0.77
Lp(a)&OxPL-apoB&OxPL-apo(a)	2.36 (1.42, 3.92)	<0.001	0.56	2.08 (1.17, 3.70)	0.01	0.77

Multivariable model: age, sex, race, systolic blood pressure, HDL-C, total cholesterol, diabetes, smoking, and history of cardiovascular disease. apoB, apolipoprotein B; apo(a), apolipoprotein(a); CI, confidence interval; HR, hazard ratio; Lp(a), lipoprotein(a); OxPL, oxidized phospholipids.

**Fig. 2:**
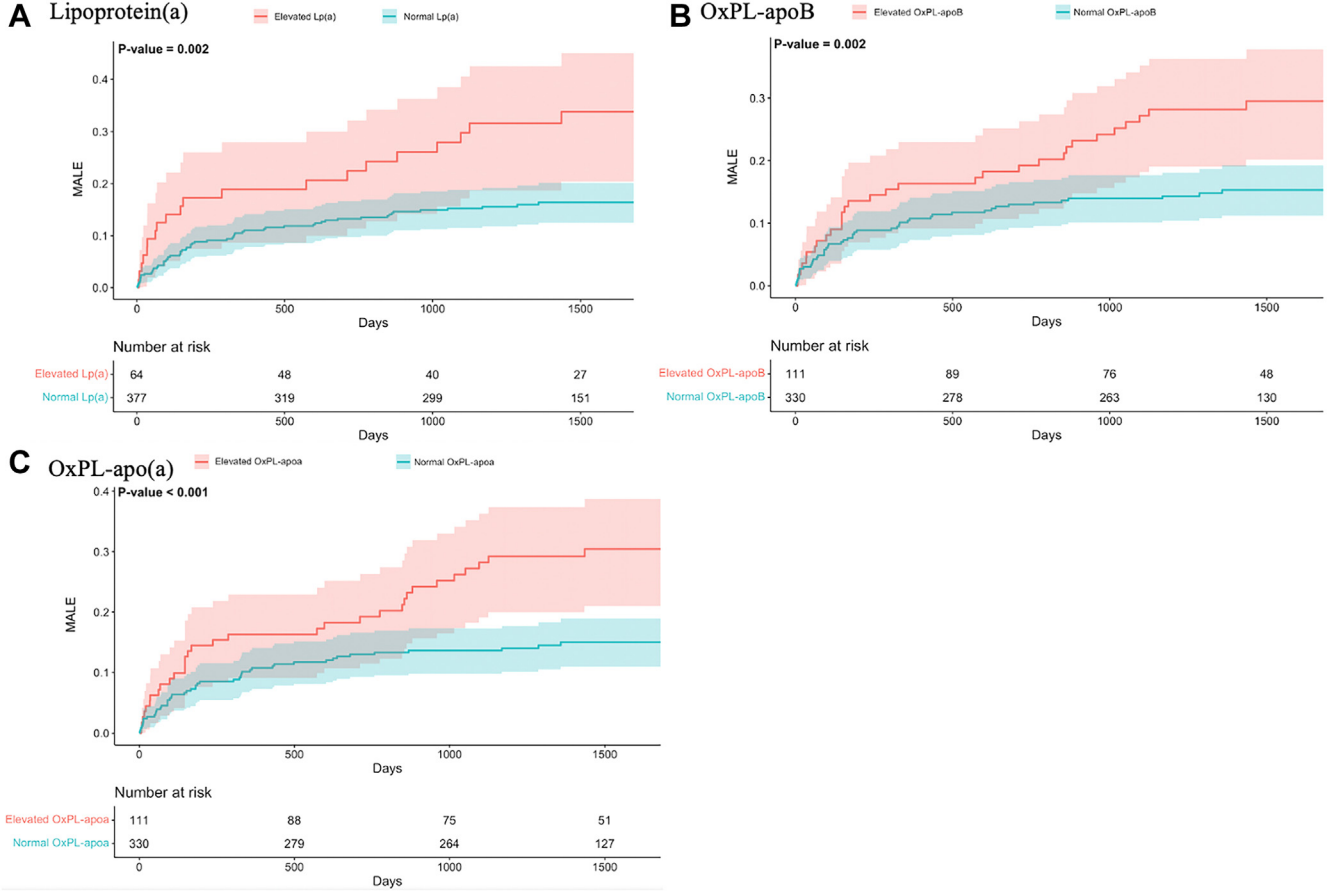
Cumulative rates of major adverse limb events (MALE) among patients with and without elevated (A) log(2) Lp(a), (B) OxPL-apoB, and (C) OxPL-apo(a) levels during follow-up. Elevated Lp(a) was defined as Lp(a) ≥150 nmol/L (≥50 mg/dl). Elevated OxPL-apoB and OxPL-apo(a) were defined based levels ≥75th percentiles (OxPL-apoB ≥8.2 nmol/L and OxPL-apo(a) ≥35.8 nmol/L).

**Fig. 3:**
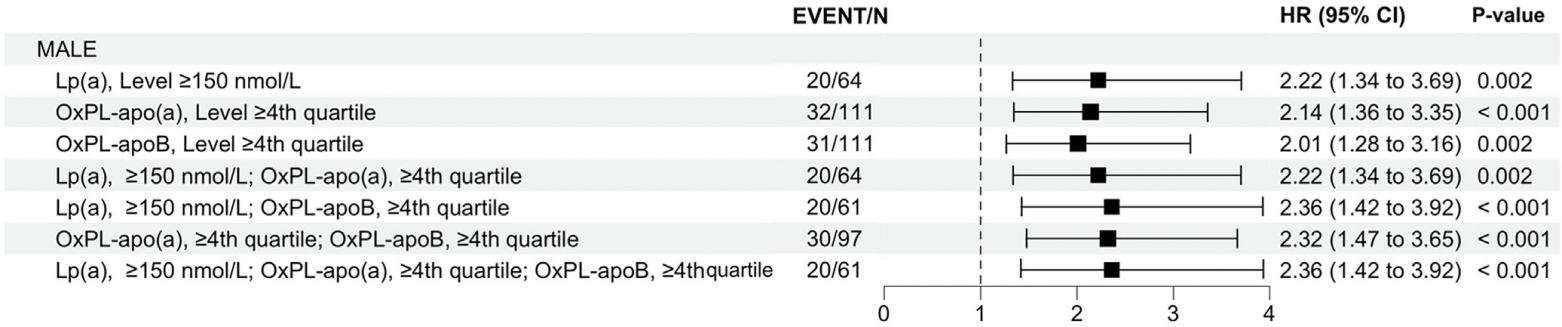
Hazard ratios of major adverse limb events in patients with elevated Lp(a), OxPL-apoB, and OxPL-apo(a) levels. Patients with elevated Lp(a) and OxPLs had the highest risk of developing adverse events.

Participants with elevated Lp(a), OxPL-apo(a), and OxPL-apoB were more likely to experience earlier MALE as evident by ETR of 0.24 (95% CI: 0.06, 0.99), 0.17 (95% CI: 0.04, 0.63), and 0.22 (95%CI: 0.06, 0.79), respectively. In cumulative hazard curves, higher values for Lp(a) (log-rank *P* < 0.002), OxPL-apoB (log-rank *P* = 0.002), and OxPL-apo(a) (log-rank *P* < 0.001) were associated with shorter time to first MALE (Fig. 2). Finally, Fig. 3 depicts HRs of MALE stratified by combinations of levels of Lp(a) and OxPLs. Participants with elevated Lp(a), OxPLs, and combinations of these levels had more than twice the risk of MALE compared to participants with normal levels for these measures.

## Discussion

In this prospective cohort of individuals who underwent coronary and/or peripheral angiography, we demonstrated participants with higher Lp(a) and related OxPLs had elevated unadjusted and adjusted odds of extracoronary vascular disease. After examining long-term adverse outcomes of extracoronary vascular disease, Lp(a) and OxPLs were predictive of future MALE. The risk of future MALE was similarly high among participants with elevated Lp(a) and combinations of elevated Lp(a) and OxPLs. These findings emphasize that Lp(a) alone captures the risk profile of Lp(a), OxPL-apoB, and OxPL-apo(a) in the development and progression of atherosclerotic plaque in peripheral arteries. Future studies should investigate the efficacy of novel therapies to lower Lp(a) in participants diagnosed with extracoronary vascular disease to prevent adverse-related outcomes.

Although the role of Lp(a) and related particles in the development of atherosclerosis and complications of coronary artery atherosclerosis is well established, the role of Lp(a) in the development of extracoronary vascular disease has been controversial in the literature (42, 43). There have been large cohort studies of healthy patients where Lp(a) levels have not been associated with extracoronary vascular disease. In a prospective cohort of 14,916 healthy adult men who were followed for development of symptomatic peripheral artery disease (PAD), (30) authors showed that higher quartiles of Lp(a) did not predict future PAD events. In a similar fashion, a study (29) of 27,935 US adult females who were followed for 12 years reported no association between Lp(a) concentrations and future extracoronary vascular disease events. However, measurement of Lp(a) in both cohorts were made around 30 years ago, and since that time, the distributions, recognition, and management of atherogenic lipoproteins and other risk factors have substantially advanced. Furthermore, both cohorts studied younger individuals and thus, despite large sizes, captured less than 200 incident PAD cases each. More contemporary studies in older populations known to be at higher risk for PAD have identified high levels of Lp(a) in patients with poor ankle/brachial index, which is a marker of PAD (44). A human genetic study of 112,338 participants also linked Lp(a) with PAD (28). Lp(a) has also been associated with the development of symptomatic extracoronary vascular disease (45, 46, 47). Prior studies have linked Lp(a) with both coronary and extracoronary vascular calcification among asymptomatic individuals (48). We showed that Lp(a) and OxPLs associate with both extracoronary vascular disease and complications of extracoronary vascular disease, namely MALE.

There is also mounting evidence that increased Lp(a) is linked to limb-related complications. Studies have reported the association between Lp(a) and need for revascularization as well as limb amputation (25, 47). Notably, among more than 16,500 patients that had an Lp(a) measured during clinical evaluation, Guedon *et al.* utilized accelerated time failure methodology similar to this analysis, finding that higher concentrations of Lp(a) were associated with MALE during follow-up (42). Our data are in concordance with these findings, as we demonstrate the importance of Lp(a) measurement in predicting limb-related complications in those with extracoronary vascular disease. In our study cohort with fewer than 100 MALE events, we were still able to show Lp(a) was associated with MALE using an adjusted accelerated failure time model. This model assumes the cumulative effect of a covariate may escalate over time, and still Lp(a) had an independent association.

This study provides novel information that Lp(a) alone captures the risk profile of Lp(a), OxPL-apoB, and OxPL-apo(a) in the development and progression of atherosclerotic plaque in peripheral arteries. OxPL formation depends heavily on the burden of oxidative stress and has been recently investigated as a biomarker in atherosclerosis (37). OxPLs are theorized to contribute to Lp(a) pathogenesis by enhancing foam cell formation and inflammatory cell recruitment (49, 50). Specifically, OxPL-apo(a) represents OxPL concentrations within Lp(a). OxPL-apoB represents OxPL concentrations within all apoB-containing lipoproteins, which include chylomicrons, VLDL. IDL, LDL, and Lp(a). This study redemonstrates a high degree of correlation between Lp(a) and OxPLs and an association between these biomarkers and our outcomes of interest. However, we do not show an independent association between these biomarkers and our outcomes of interest (39). Multivariate models including covariates and Lp(a) for both extracoronary vascular disease and MALE show an unchanged or lower C-statistic with the serial addition of OxPLs, indicating multicollinearity issues that reduce predictive accuracy. Although higher concentrations of all three particles measured (Lp(a), OxPL-apo(a), and OxPL-apoB) were associated with shorter time to first MALE event in both ETR and cumulative hazard analyses, the addition of OxPLs did not enhance the association and therefore are not necessary to obtain when Lp(a) levels are readily available.

Our results demonstrate a correlation between Lp(a) and extracoronary vascular disease, which is not substantially enhanced with the addition of OxPLs. Reassuringly, there are Lp(a) assays readily available to clinicians, while assays for OxPL-apoB and OxPL-apo(a) are more difficult to access (51, 52). These available tests for Lp(a) may be sufficient for quantifying the risk of in the development and progression of atherosclerotic plaque in peripheral arteries. This correlation also supports further investigation of Lp(a) as a potential biomarker of extracoronary vascular disease. Lp(a) concentrations are highly heritable, with minimal influence by diet or environmental factors (53). Until recently, medical therapies were lacking for substantial Lp(a) lowering. With the development of treatments such as antisense oligonucleotide therapy, the prospect now exists to substantially lower Lp(a) (54). The findings of this analysis make a strong case to explore effect of such therapies to lower Lp(a) in patients with extracoronary vascular disease with a goal to slow progression of disease and reduce major complications of the disease such as need for revascularization or amputation.

Our study has several limitations. First, majority of study participants were white males. Lp(a) is predominantly heritable with higher concentrations in Black individuals (55, 56); thus, our results may not be generalizable and should not be extended to a more multiethnic/multiracial population with more female representation. Second, in order to provide statistical power for the analysis, we constructed a comparator control group using an approach that minimizes the risk for unrecognized extracoronary vascular disease through exclusion of those with prevalent or incident clinical extracoronary vascular disease, and who were also without angiographic CAD. Despite this approach, it is possible some of the control participants had occult extracoronary vascular disease. Even with these efforts, the sample size is relatively small, and with multivariable adjustment, associations between Lp(a) and OxPLs were attenuated owing to wider CI around an OR that was comparable to unadjusted analyses; larger studies using these assays are now justified. Finally, study participants were followed up for median of 3.7 years; future studies may assess the impact of elevated Lp(a) on longer term limb-related complications.

## Data availability

These data can be shared for research upon request from the corresponding author, Dr Pradeep Natarajan, MD, MMSc (Email: pnatarajan@mgh.harvard).

## Supplemental data

This article contains supplemental data.

## Conflict of interest

This study was sponsored by 10.13039/100004336Novartis as an investigator-initiated study. The sponsor provided feedback on the study design but not data interpretation. X. H., J. R. C., and A. B. are employees of Novartis. S. T. is a coinventor and receives royalties from patents owned by University of California San Diego (UCSD) and is a cofounder and has an equity interest in Oxitope, LLC and its affiliates, Kleanthi Diagnostics, LLC and Covicept Therapeutics, Inc and has a dual appointment at UCSD and Ionis Pharmaceuticals. The terms of this arrangement have been reviewed and approved by the University of California, San Diego, in accordance with its conflict-of-interest policies. J. L. J. is a Trustee of the American College of Cardiology, a Board member of Imbria Pharmaceuticals, a Director at Jana Care, has received grant support from Applied Therapeutics, Innolife, Novartis Pharmaceuticals, and 10.13039/100014386Abbott Diagnostics, consulting income from 10.13039/100000046Abbott, 10.13039/100014554Janssen, 10.13039/100004336Novartis, Prevencio, and Roche Diagnostics and participates in clinical endpoint committees/data safety monitoring boards for Abbott, AbbVie, Bayer, CVRx, Siemens, and Takeda. P.N. reports research grants from Allelica, 10.13039/100002429Amgen, 10.13039/100017567Apple, 10.13039/100008497Boston Scientific, 10.13039/100004328Genentech, Rochee, and 10.13039/100004336Novartis, personal fees from Allelica, 10.13039/100017567Apple, 10.13039/100004325AstraZeneca, Blackstone Life Sciences, Creative Education Concepts, CRISPR Therapeutics, 10.13039/100004312Eli Lilly & Co, Foresite Labs, 10.13039/100001127Genentech, Rochee, GV, HeartFlow, Magnet Biomedicine, 10.13039/100004334Merck, and 10.13039/100004336Novartis, scientific advisory board membership of Esperion Therapeutics, Preciseli, and TenSixteen Bio, scientific co-founder of TenSixteen Bio, equity in MyOme, Preciseli, and TenSixteen Bio, and spousal employment at Vertex Pharmaceuticals, all unrelated to the present work. The remaining authors have nothing to disclose. Although these relationships have been identified for conflict-of-interest management based on the project's overall scope, the research findings included in this particular publication may not necessarily relate to the interests of the above companies.

## References

[1] Lin J., Chen Y., Jiang N., Li Z., Xu S. (2022). Burden of peripheral artery disease and its attributable risk factors in 204 countries and territories from 1990 to 2019. Front. Cardiovasc. Med..

[2] Hirsch A.T., Duval S. (2013). The global pandemic of peripheral artery disease. Lancet.

[3] Feigin V.L. (2019). Anthology of stroke epidemiology in the 20th and 21st centuries: assessing the past, the present, and envisioning the future. Int. J. Stroke.

[4] Lineback C.M., Stamm B., Sorond F., Caprio F.Z. (2023). Carotid disease, cognition, and aging: time to redefine asymptomatic disease?. Geroscience.

[5] Savji N., Rockman C.B., Skolnick A.H., Guo Y., Adelman M.A., Riles T. (2013). Association between advanced age and vascular disease in different arterial territories: a population database of over 3.6 million subjects. J. Am. Coll. Cardiol..

[6] Virani S.S., Alonso A., Aparicio H.J., Benjamin E.J., Bittencourt M.S., Callaway C.W. (2021). Heart disease and stroke statistics - 2021 update: a report from the American heart association. Circulation.

[7] Bentzon J.F., Otsuka F., Virmani R., Falk E. (2014). Mechanisms of plaque formation and rupture. Circ. Res..

[8] Bevan G.H., White Solaru K.T. (2020). Evidence-based medical management of peripheral artery disease. Arterioscler. Thromb. Vasc. Biol..

[9] Armengol G., Goudot G., Miranda S., Benhamou Y., Tafflet M., Guillet H. (2023). Symptomatic upper extremity peripheral artery disease is associated with poor outcomes and a broad spectrum of etiologies. Angiology.

[10] Safak E., Wilke C., Derer W., Busjahn A., Gross M., Moeckel M. (2013). Long-term follow-up of patients with atherosclerotic renal artery disease. J. Am. Soc. Hypertens..

[11] Fortington L.V., Geertzen J.H.B., Van Netten J.J., Postema K., Rommers G.M., Dijkstra P.U. (2013). Short and long term mortality rates after a lower limb amputation. Eur. J. Vasc. Endovasc Surg..

[12] Hicks C.W., Clark T.W.I., Cooper C.J., de Bhailís Á.M., De Carlo M., Green D. (2022). Atherosclerotic renovascular disease: a KDIGO (kidney disease: improving global outcomes) controversies conference. Am. J. Kidney Dis..

[13] Conte M.S., Pomposelli F.B., Clair D.G., Clair D.G., Geraghty P.J., McKinsey J.F. (2015). Society for Vascular Surgery practice guidelines for atherosclerotic occlusive disease of the lower extremities: management of asymptomatic disease and claudication. J. Vasc. Surg..

[14] AbuRahma A.F., Avgerinos E.D., Chang R.W., Darling R.C., Duncan A.A., Forbes T.L. (2022). Society for Vascular Surgery clinical practice guidelines for management of extracranial cerebrovascular disease. J. Vasc. Surg..

[15] Berliner J.A., Navab M., Fogelman A.M., Frank J.S., Demer L.L., Edwards P.A. (1995). Atherosclerosis: basic mechanisms. Circulation.

[16] Linton M.F., Yancey P.G., Davies S.S. (2019). The role of lipids and lipoproteins in atherosclerosis. Science.

[17] Kochar A., Mulder H., Rockhold F.W., Baumgartner I., Berger J.S., Blomster J.I. (2020). Cause of death among patients with peripheral artery disease: insights from the EUCLID trial. Circ. Cardiovasc. Qual. Outcomes.

[18] Schwartz G.G., Steg P.G., Szarek M., Bittner V.A., Diaz R., Goodman S.G. (2020). Peripheral artery disease and venous thromboembolic events after acute coronary syndrome: role of lipoprotein(a) and modification by alirocumab: prespecified analysis of the Odyssey outcomes randomized clinical trial. Circulation.

[19] Tselepis A.D. (2018). Oxidized phospholipids and lipoprotein-associated phospholipase A2 as important determinants of Lp(a)functionality and pathophysiological role. J. Biomed. Res..

[20] Miles L.A., Fless G.M., Levin E.G., Scanu A.M., Plow E.F. (1989). A potential basis for the thrombotic risks associated with lipoprotein(a). Nature.

[21] Nielsen L.B., Stender S., Kjeldsen K., Nordestgaard B.G. (1996). Specific accumulation of lipoprotein(a) in balloon-injured rabbit aorta in vivo. Circ. Res..

[22] Nielsen L.B., Nordestgaard B.G., Stender S., Niendorf A., Kjeldsen K. (1995). Transfer of lipoprotein(a) and LDL into aortic intima in normal and in cholesterol-fed rabbits. Arterioscler. Thromb. Vasc. Biol..

[23] Tzoulaki I., Murray G.D., Lee A.J., Rumley A., Lowe G.D.O., Fowkes F.G.R. (2007). Inflammatory, haemostatic, and rheological markers for incident peripheral arterial disease: Edinburgh Artery Study. Eur. Heart J..

[24] Laschkolnig A., Kollerits B., Lamina C., Meisinger C., Rantner B., Stadler M. (2014). Lipoprotein (a) concentrations, apolipoprotein (a) phenotypes, and peripheral arterial disease in three independent cohorts. Cardiovasc. Res..

[25] Sanchez Muñoz-Torrero J.F., Rico-Martín S., Álvarez L.R., Aguilar E., Alcalá J.N., Monreal M. (2018). Lipoprotein (a) levels and outcomes in stable outpatients with symptomatic artery disease. Atherosclerosis.

[26] Gurdasani D., Sjouke B., Tsimikas S., Hovingh G.K., Luben R.N., Wainwright N.W.J. (2012). Lipoprotein(a) and risk of coronary, cerebrovascular, and peripheral artery disease: the EPIC-Norfolk prospective population study. Arterioscler. Thromb. Vasc. Biol..

[27] Golledge J., Rowbotham S., Velu R., Quigley F., Jenkins J., Bourke M. (2020). Association of serum lipoprotein (a) with the requirement for a peripheral artery operation and the incidence of major adverse cardiovascular events in people with peripheral artery disease. J. Am. Heart Assoc..

[28] Emdin C.A., Khera A.V., Natarajan P., Klarin D., Won H.H., Peloso G.M. (2016). Phenotypic characterization of genetically lowered human lipoprotein(a) levels. J. Am. Coll. Cardiol..

[29] Pradhan A.D., Shrivastava S., Cook N.R., Rifai N., Creager M.A., Ridker P.M. (2008). Symptomatic peripheral arterial disease in women: nontraditional biomarkers of elevated risk. Circulation.

[30] Ridker P.M., Stampfer M.J., Rifai N. (2001). Novel risk factors for systemic atherosclerosis: a comparison of C-reactive protein, fibrinogen, homocysteine, lipoprotein(a), and standard cholesterol screening as predictors of peripheral arterial disease. JAMA.

[31] Gaggin H.K., Liu Y., Lyass A., van Kimmenade R.R.J., Motiwala S.R., Kelly N.P. (2017). Incident type 2 myocardial infarction in a cohort of patients undergoing coronary or peripheral arterial angiography. Circulation.

[32] McCarthy C.P., Ibrahim N.E., van Kimmenade R.R.J., Gaggin H.K., Simon M.L., Gandhi P. (2018). A clinical and proteomics approach to predict the presence of obstructive peripheral arterial disease: from the Catheter Sampled Blood Archive in Cardiovascular Diseases (CASABLANCA) Study. Clin. Cardiol..

[33] Joosten M.M., Pai J.K., Bertoia M.L., Rimm E.B., Spiegelman D., Mittleman M.A. (2012). Associations between conventional cardiovascular risk factors and risk of peripheral artery disease in men. JAMA.

[34] Marcovina S.M., Navabi N., Allen S., Gonen A., Witztum J.L., Tsimikas S. (2022). Development and validation of an isoform-independent monoclonal antibody-based ELISA for measurement of lipoprotein(a). J. Lipid Res..

[35] Marcovina S.M., Albers J.J. (2016). Lipoprotein (a) measurements for clinical application. J. Lipid Res..

[36] Tsimikas S., Lau H.K., Han K.R., Shortal B., Miller E.R., Segev A. (2004). Percutaneous coronary intervention results in acute increases in oxidized phospholipids and lipoprotein(a): short-term and long-term immunologic responses to oxidized low-density lipoprotein. Circulation.

[37] Bertoia M.L., Pai J.K., Lee J.H., Taleb A., Joosten M.M., Mittleman M.A. (2013). Oxidation-specific biomarkers and risk of peripheral artery disease. J. Am. Coll. Cardiol..

[38] Tsimikas S., Witztum J.L. (2024). Oxidized phospholipids in cardiovascular disease. Nat. Rev. Cardiol..

[39] Gilliland T.C., Liu Y., Mohebi R., Miksenas H., Haidermota S., Wong M. (2023). Lipoprotein(a), oxidized phospholipids, and coronary artery disease severity and outcomes. J. Am. Coll. Cardiol..

[40] Grundy S.M., Stone N.J., Bailey A.L., Beam C., Birtcher K.K., Blumenthal R.S. (2019). 2018 AHA/ACC/AACVPR/AAPA/ABC/ACPM/ADA/AGS/APhA/ASPC/NLA/PCNA guideline on the management of blood cholesterol: executive summary: a report of the American College of Cardiology/American heart association task force on clinical practice guidelines. J. Am. Coll. Cardiol..

[41] Matsushita K., Sang Y., Ning H., Ballew S.H., Chow E.K., Grams M.E. (2019). Lifetime risk of lower-extremity peripheral artery disease defined by ankle-brachial index in the United States. J. Am. Heart Assoc..

[42] Guédon A.F., De Freminville J.B., Mirault T., Mohamedi N., Rance B., Fournier N. (2022). Association of lipoprotein(a) levels with incidence of major adverse limb events. JAMA Netw. Open.

[43] Duarte L.F., Giugliano R.P. (2022). Lipoprotein(a) and its significance in cardiovascular disease: a review. JAMA Cardiol..

[44] Volpato S., Vigna G.B., McDermott M.M., Cavalieri M., Maraldi C., Lauretani F. (2010). Lipoprotein[a], inflammation, and peripheral arterial disease in a community-based sample of older men and women (the InCHIANTI study). Am. J. Cardiol..

[45] Rehberger Likozar A., Zavrtanik M., Šebeštjen M. (2020). Lipoprotein(a) in atherosclerosis: from pathophysiology to clinical relevance and treatment options. Ann. Med..

[46] Ohira T., Schreiner P.J., Morrisett J.D., Chambless L.E., Rosamond W.D., Folsom A.R. (2006). Lipoprotein(a) and incident ischemic stroke: the Atherosclerosis Risk in Communities (ARIC) study. Stroke.

[47] Thomas P.E., Vedel-Krogh S., Nielsen S.F., Nordestgaard B.G., Kamstrup P.R. (2023). Lipoprotein(a) and risks of peripheral artery disease, abdominal aortic aneurysm, and major adverse limb events. J. Am. Coll. Cardiol..

[48] Obisesan O.H., Kou M., Wang F.M., Boakye E., Honda Y., Uddin S.M.I. (2022). Lipoprotein(a) and subclinical vascular and valvular calcification on cardiac computed tomography: the atherosclerosis risk in communities study. J. Am. Heart Assoc..

[49] Boffa M.B., Koschinsky M.L. (2019). Oxidized phospholipids as a unifying theory for lipoprotein(a) and cardiovascular disease. Nat. Rev. Cardiol..

[50] Van Der Valk F.M., Bekkering S., Kroon J., Yeang C., Van den Bossche J., van Buul J.D. (2016). Oxidized phospholipids on lipoprotein(a) elicit arterial wall inflammation and an inflammatory monocyte response in humans. Circulation.

[51] Bhatia H.S., Yeang C., Baruch A., Yang X., Stroes E.S.G., Tsimikas S. (2021). PCSK9 inhibition and oxidized phospholipids. J. Am. Coll. Cardiol..

[52] Tsimikas S. (2019). Potential causality and emerging medical therapies for lipoprotein(a) and its associated oxidized phospholipids in calcific aortic valve stenosis. Circ. Res..

[53] Tsimikas S. (2017). A test in context: lipoprotein(a): diagnosis, prognosis, controversies, and emerging therapies. J. Am. Coll. Cardiol..

[54] Yeang C., Karwatowska-Prokopczuk E., Su F., Dinh B., Xia S., Witztum J.L. (2022). Effect of pelacarsen on lipoprotein(a) cholesterol and corrected low-density lipoprotein cholesterol. J. Am. Coll. Cardiol..

[55] Guan W., Cao J., Steffen B.T., Post W.S., Stein J.H., Tattersall M.C. (2015). Race is a key variable in assigning lipoprotein(a) cutoff values for coronary heart disease risk assessment: the Multi-Ethnic Study of Atherosclerosis. Arterioscler. Thromb. Vasc. Biol..

[56] Virani S.S., Brautbar A., Davis B.C., Nambi V., Hoogeveen R.C., Sharrett A.R. (2012). Associations between lipoprotein(a) levels and cardiovascular outcomes in black and white subjects: the Atherosclerosis Risk in Communities (ARIC) Study. Circulation.

