# Supplementary material for: Associations between lipoprotein(a), oxidized phospholipids, and extracoronary vascular disease
Source: J Lipid Res. 2024 Jun 26;65(7):100585. doi: 10.1016/j.jlr.2024.100585 (PMC11298641; doi:10.1016/j.jlr.2024.100585)
Supplement: Supplemental Materials [file mmc1.docx]

**Associations between lipoprotein (a), oxidized phospholipids, and peripheral arterial disease**

**Brief title**: Lipoprotein(a), oxidized phospholipids and PAD

| **Content** | **Page** |
| --- | --- |
| **Supplementary Table 1**. Threshold analysis for the ability of Lp(a) and OxPLs to discriminate extra-coronary vascular disease. | 2 |
| **Supplementary Table 2**. Baseline characteristics of participants according to Lp (a) level. | 3 |
| **Supplementary Table 3**. Distribution of OxPL levels across Lp(a) quartiles. | 4 |
| **Supplementary Table 4**. Association of Lp(a) and additional OxPLs with extra-coronary vascular disease. | 5 |
| **Supplementary Table 5**. Association of OxPLs with extra-coronary vascular disease among all participants and MALE among participants with extra-coronary vascular disease. | 6 |
| **Supplementary Table 6.** Association of Lp(a) and additional OxPLs with major adverse limb event in participants with extra-coronary vascular disease. | 7 |
| **Supplementary Figure 1.** Flow diagram of participants analyzed in this study. | 8 |
| **Supplementary Figure 2.** Correlation heatmap between Lp(a), OxPLs, and traditional lipid parameters. | 9 |

**Supplementary Table 1**. Threshold analysis for the ability of Lp(a) and OxPLs to discriminate extra-coronary vascular disease.

|  | **Cut point** | **Sensitivity (%)** | **Specificity**  **(%)** | **Youden Index** |
| --- | --- | --- | --- | --- |
| Lp(a) | 139 nmol/L | 32 | 87 | 0.18 |
| OxPL-Apo(a) | 37.7 nmol/L | 41 | 80 | 0.21 |
| OxPL-ApoB | 9.5 nmol/L | 38 | 82 | 0.20 |

**Supplementary Table 2**. Baseline characteristics of study participants according to Lp (a) level.

|  | **Normal Lp(a)**  **(n=362)** | **Elevated Lp(a)**  **(n=79)** | **p-value** |
| --- | --- | --- | --- |
| Age (mean (SD)) | 64.41 (11.65) | 65.00 (11.23) | 0.68 |
| Male (%) | 224 (61.9) | 45 (57.0) | 0.49 |
| Caucasian (%) | 327 (90.3) | 75 (94.9) | 0.56 |
| **Medical Conditions (%)** |  |  |  |
| Hypertension | 252 (69.6) | 66 (83.5) | 0.02 |
| Dyslipidemia | 198 (54.7) | 58 (73.4) | 0.003 |
| Coronary artery disease | 120 (33.1) | 41 (51.9) | 0.003 |
| Prior MI | 42 (11.6) | 18 (22.8) | 0.01 |
| Heart Failure | 75 (20.7) | 27 (34.2) | 0.02 |
| COPD | 71 (19.6) | 21 (26.6) | 0.22 |
| Diabetes Mellitus | 84 (23.2) | 24 (30.4) | 0.23 |
| CVA/TIA | 36 (9.9) | 14 (17.7) | 0.08 |
| Chronic kidney disease | 34 (9.4) | 12 (15.2) | 0.19 |
| Renal replacement therapy | 4 (1.1) | 3 (3.8) | 0.22 |
| Smoker | 46 (12.8) | 16 (20.8) | 0.10 |
| Afib/Aflutter | 80 (22.1) | 15 (19.0) | 0.65 |
| Prior angioplasty | 47 (13.0) | 13 (16.5) | 0.53 |
| Prior CABG | 26 (7.2) | 15 (19.0) | 0.002 |
| Prior PCI | 62 (17.1) | 23 (29.1) | 0.02 |
| **Medications (%)** |  |  |  |
| ACEi/ARB | 181 (50.4) | 49 (62.0) | 0.08 |
| β-blocker | 216 (60.0) | 49 (62.0) | 0.84 |
| Aldosterone antagonist | 17 (4.7) | 3 (3.8) | 0.95 |
| Loop diuretic | 78 (21.7) | 18 (22.8) | 0.95 |
| Nitrate | 39 (10.8) | 13 (16.5) | 0.23 |
| Calcium channel blocker | 85 (23.7) | 22 (27.8) | 0.52 |
| Statin | 224 (62.2) | 64 (81.0) | 0.002 |
| Aspirin | 245 (68.1) | 60 (75.9) | 0.21 |
| Warfarin | 72 (20.1) | 11 (13.9) | 0.27 |
| Clopidogrel | 64 (17.8) | 19 (24.1) | 0.26 |
| **Laboratory data (median [IQR])** |  |  |  |
| Sodium | 140 (138, 142) | 140 (137, 141) | 0.18 |
| BUN | 17 (14, 23) | 20 (16, 29) | 0.01 |
| Creatinine | 1.06 (0.89, 1.26) | 1.12 (0.92, 1.49) | 0.10 |
| Total cholesterol | 152 (129, 182) | 155 (133, 200) | 0.63 |
| LDL-C | 85.00 (62.00, 107.75) | 76.50 (58.00, 112.75) | 0.73 |
| Glycohemoglobin | 6.00 (5.45, 6.90) | 5.95 (5.65, 6.68) | 0.77 |
| Glucose | 100.50 (90.00, 117.25) | 103.00 (90.50, 136.00) | 0.27 |
| Hemoglobin | 13.30 (12.10, 14.57) | 12.80 (11.60, 14.10) | 0.05 |
| Lp (a) | 20.03 (9.82, 40.22) | 210.53 (154.10, 263.94) | <0.001 |
| OxPL-Apo(a) | 8.03 (4.07, 17.68) | 54.09 (46.02, 61.26) | <0.001 |
| OxPL-ApoB | 3.27 (2.63, 4.61) | 13.44 (11.59, 17.31) | <0.001 |

Abbreviations: MI: myocardial infarction, CVA: cerebrovascular accident, TIA: transient ischemic event, Afib: atrial fibrillation, CABG: coronary artery bypass graft, PCI: percutaneous coronary intervention, ACEi: angiotensin converting enzyme inhibitor, ARB: angiotensin receptor blocker, CCB: calcium channel blocker, BUN: blood urea nitrogen, LDL-C: low density lipoprotein cholesterol, Lp(a): lipoprotein(a): OxPL: oxidized phospholipids.

**Supplementary Table 3.** Distribution of OxPL levels across Lp(a) quartiles.

|  | **Lp(a) quartile 1** | **Lp(a) quartile 2** | **Lp(a) quartile 3** | **Lp(a) quartile 4** | **p-value** |
| --- | --- | --- | --- | --- | --- |
| Participants (n) | 111 | 110 | 110 | 110 |  |
| Lp(a) median [IQR] | 6.23 [4.52, 8.93] | 17.47 [14.03, 21.77] | 41.19 [32.69, 55.35] | 160.65 [117.29, 243.28] | <0.001 |
| OxPL-apo(a) median [IQR] | 2.99 [1.99, 4.50] | 6.66 [4.89, 10.22] | 18.92 [13.08, 28.52] | 48.81 [41.69, 58.55] | <0.001 |
| OxPL-apoB median [IQR] | 2.57 [2.17, 2.84] | 3.01 [2.62, 3.56] | 4.60 [3.87, 5.80] | 12.65 [9.73, 15.56] | <0.001 |

**Supplementary Table 4**. Association of Lp(a) and additional OxPLs with extra-coronary vascular disease.

|  | Multivariable model | |  |
| --- | --- | --- | --- |
|  | OR (95%CI) | p-value | C-statistic |
| Model 1: Lp(a), covariates |  |  | 0.82 |
| Lp(a) Log_(2)_ | 1.15 (0.97, 1.36) | 0.11 |  |
| Model 2: Lp(a), covariates, OxPL-apoB log |  |  | 0.82 |
| Lp(a) Log_(2)_ | 1.25 (0.94, 1.82) | 0.17 |  |
| OxPL-apoB Log_(2)_ | 0.80 (0.39, 1.82) | 0.51 |  |
| Model 3: Lp(a), covariates, OxPL-apoB threshold |  |  | 0.82 |
| Lp(a) ≥150 | 2.71 (1.04, 7.25) | 0.04 |  |
| OxPL-apoB ≥8.2 nmol/L | 0.75 (0.35, 1.58) | 0.46 |  |
| Model 4: Lp(a), covariates, OxPL-apo(a) log |  |  | 0.82 |
| Lp(a) Log_(2)_ | 1.66 (1.09, 3.01) | 0.06 |  |
| OxPL-apo(a) Log_(2)_ | 0.60 (0.31, 1.00) | 0.09 |  |
| Model 5: Lp(a), covariates, OxPL-apo(a) threshold |  |  | 0.82 |
| Lp(a) ≥150 | 1.87 (0.70, 5.11) | 0.22 |  |
| OxPL-apo(a) ≥35.8 nmol/L | 1.17 (0.52, 2.56) | 0.69 |  |
| Model 6: Lp(a), covariates, OxPL-apo(a), OxPL-apoB log |  |  | 0.82 |
| Lp(a) Log_(2)_ | 1.55 (1.03, 2.86) | 0.09 |  |
| OxPL-apo(a) Log_(2)_ | 0.53 (0.26, 0.99) | 0.06 |  |
| OxPL-apoB Log_(2)_ | 1.46 (0.57, 3.72) | 0.43 |  |
| Model 7: Lp(a), covariates, OxPL-apo(a), OxPL-apoB threshold |  |  | 0.82 |
| Lp(a) ≥150 | 2.19 (0.79, 6.19) | 0.13 |  |
| OxPL-apo(a) ≥35.8 nmol/L | 2.01 (0.66, 6.05) | 0.21 |  |
| OxPL-apoB ≥8.2 nmol/L | 0.48 (0.16, 1.34) | 0.17 |  |

All models include the covariates of age, sex, race, systolic blood pressure, HDL-c, total cholesterol, diabetes, smoking, and history of cardiovascular disease.

**Supplemental Table 5.** Association of OxPLs with extra-coronary vascular disease among all participants and MALE among participants with extra-coronary vascular disease.

|  | Extra-coronary vascular disease | | MALE | |
| --- | --- | --- | --- | --- |
|  | OR (95%CI) | p-value | OR (95%CI) | p-value |
| OxPL-apoB Log_(2)_, per 1 unit increment |  |  |  |  |
| All Patients | 1.36 (1.02, 1.81) | 0.03 | 1.50 (1.11, 2.03) | 0.008 |
| Lp(a) <25^th^ percentile | 0.41 (0.07, 2.16) | 0.29 | 0.67 (0.10, 4.42) | 0.68 |
| OxPL-apo(a) Log_(2)_, per 1 unit increment |  |  |  |  |
| All Patients | 1.11 (0.94, 1.33) | 0.23 | 1.25 (1.02, 1.53) | 0.003 |
| Lp(a) <25^th^ percentile | 0.76 (0.32, 1.55) | 0.39 | 1.18 (0.45, 3.06) | 0.74 |

**Supplementary Table 6**. Association of Lp(a) and additional OxPLs with major adverse limb event in participants with extra-coronary vascular disease.

|  | Multivariable model | |  |
| --- | --- | --- | --- |
|  | HR (95%CI) | p-value | C-statistic |
| Model 1: Lp(a), covariates |  |  | 0.77 |
| Lp(a) Log_(2)_ | 1.21 (1.01, 1.45) | 0.04 |  |
| Model 2: Lp(a), covariates, OxPL-apoB log |  |  | 0.58 |
| Lp(a) Log_(2)_ | 1.43 (0.82, 2.49) | 0.21 |  |
| OxPL-apoB Log_(2)_ | 0.77 (0.31, 1.93) | 0.58 |  |
| Model 3: Lp(a), covariates, OxPL-apoB threshold |  |  | 0.57 |
| Lp(a) ≥150 | 1.77 (0.72, 4.34) | 0.21 |  |
| OxPL-apoB ≥10.84 nmol/L | 1.22 (0.54, 2.76) | 0.63 |  |
| Model 4: Lp(a), covariates, OxPL-apo(a) log |  |  | 0.57 |
| Lp(a) Log_(2)_ | 1.30 (0.70, 2.41) | 0.41 |  |
| OxPL-apo(a) Log_(2)_ | 0.94 (0.47, 1.89) | 0.87 |  |
| Model 5: Lp(a), covariates, OxPL-apo(a) threshold |  |  | 0.58 |
| Lp(a) ≥150 | 1.19 (0.44, 3.24) | 0.73 |  |
| OxPL-apo(a) ≥42.51nmol/L | 1.95 (0.75, 5.06) | 0.17 |  |
| Model 6: Lp(a), covariates, OxPL-apo(a), OxPL-apoB log |  |  | 0.59 |
| Lp(a) Log_(2)_ | 1.44 (0.69, 3.01) | 0.33 |  |
| OxPL-apo(a) Log_(2)_ | 0.99 (0.48, 2.03) | 0.97 |  |
| OxPL-apoB Log_(2)_ | 0.77 (0.30, 1.99) | 0.59 |  |
| Model 7: Lp(a), covariates, OxPL-apo(a), OxPL-apoB threshold |  |  | 0.58 |
| Lp(a) ≥150 | 1.22 (0.44, 3.36) | 0.71 |  |
| OxPL-apo(a) ≥42.51nmol/L | 2.22 (0.65, 7.51) | 0.21 |  |
| OxPL-apoB ≥10.84nmol/L | 0.85 (0.32, 2.27) | 0.74 |  |

All models include the covariates of age, sex, race, systolic blood pressure, HDL-c, total cholesterol, diabetes, smoking, and history of cardiovascular disease.

**Supplementary Figure 1.** Flow diagram of participants analyzed in this study.

**
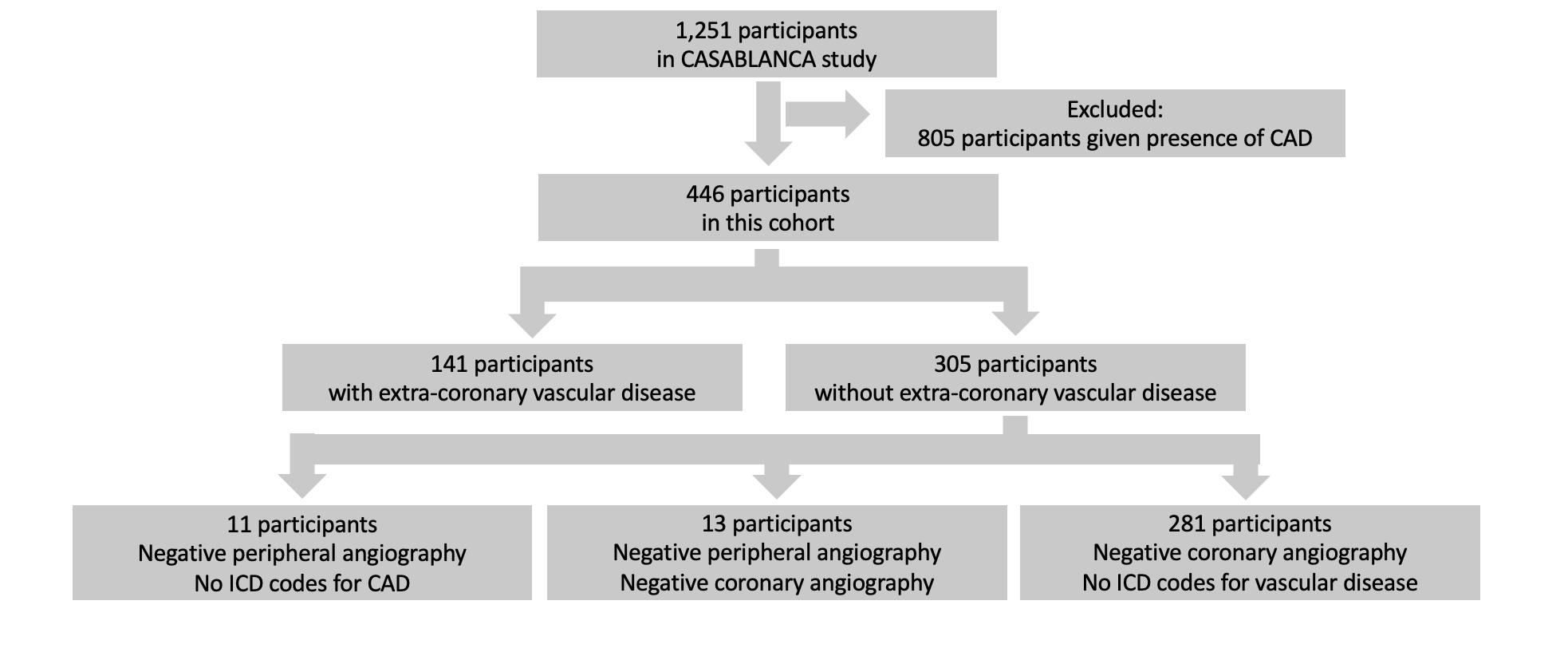
**

**Supplementary Figure 2**. Correlation heatmap between Lp(a), OxPLs, and traditional lipid parameters.


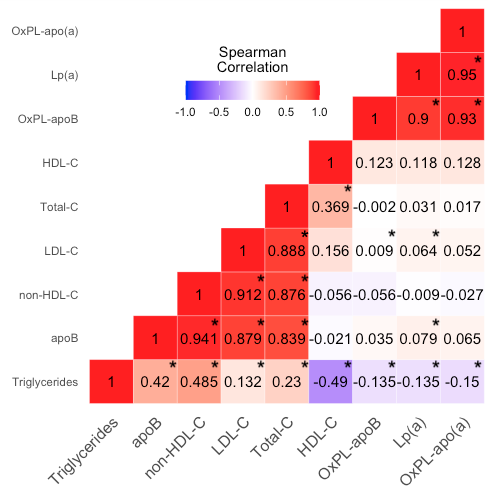


Spearman correlations between log_2_ transformed Lp(a), OxPL-apo(a), OxPL-apoB, and Lp(a) related biomarkers. Abbreviations: apo(a): apoplipoprotein a, apoB: apoplipoprotein B, LDL-C: low density lipoprotein cholesterol, HDL-C: high density lipoprotein cholesterol, Lp(a): lipoprotein(a): OxPL: oxidized phospholipids.
